# Oxidative Stress and Antioxidant Defense During Liver Regeneration After Acetaminophen Toxicity: The Preventive Potential of the Microalga *Desmodesmus armatus*

**DOI:** 10.3390/antiox15040492

**Published:** 2026-04-15

**Authors:** Halyna P. Kopylchuk, Ivanna M. Nykolaichuk, Mariia S. Ursatyi, Larysa M. Cheban, Oleksii Skorokhod, Oksana M. Voloshchuk

**Affiliations:** 1 Educational and Scientific Institute of Biology, Chemistry and Natural Resources, Yuriy Fedkovych Chernivtsi National University, 58012 Chernivtsi, Ukraine; g.kopilchuk@chnu.edu.ua (H.P.K.); i.nykolaichuk@chnu.edu.ua (I.M.N.); l.cheban@chnu.edu.ua (L.M.C.); o.voloschuk@chnu.edu.ua (O.M.V.); 2 Department of Life Sciences and Systems Biology, University of Turin, via Accademia Albertina 13, 10123 Turin, Italy

**Keywords:** acetaminophen, reactive oxygen species, superoxide dismutase, glutatione peroxidase, C-reactive protein, serum alanine aminotransferase (ALT), serum aspartate aminotransferase (AST), partial hepatectomy, toxic liver injury, microalgae *Desmodesmus armatus*

## Abstract

Liver regeneration after partial hepatectomy (PH) is critically influenced by redox balance, which may be severely disrupted under drug-induced liver injury. This study evaluated oxidative stress parameters and inflammatory markers in rats subjected to 70% PH following acetaminophen (APAP)-induced toxicity and assessed the preventive effect of the microalga *Desmodesmus armatus*. Reactive oxygen species (superoxide anion, hydroxyl radical, and hydrogen peroxide), antioxidant enzyme activities (superoxide dismutase and glutathione peroxidase), serum aminotransferases, bilirubin, and C-reactive protein were analyzed 0–168 h post-hepatectomy. APAP intoxication markedly increased mitochondrial ROS production, suppressed mitochondrial antioxidant enzyme activity, and prolonged elevations of ALT, AST, bilirubin, and CRP, accompanied by severe histological damage. Preventive administration of *D. armatus* suspension (10 mL/kg body weight at 1.5 × 10^6^ and 1.5 × 10^7^ cells/mL) attenuated oxidative stress in a dose-dependent manner. It significantly reduced ROS levels, restored mitochondrial antioxidant defenses, decreased cytolytic and cholestatic markers, and mitigated systemic inflammation. Overall, *D. armatus* exhibited hepatoprotective and redox-modulating properties, which may contribute to a more favorable microenvironment for liver recovery under toxic conditions. These findings highlight the potential of microalgae-based interventions as supportive strategies for reducing liver injury and improving recovery following acute liver injury.

## 1. Introduction

The liver is a key homeostatic organ with a broad functional profile. One of its pivotal roles—namely, the compensation of metabolic disturbances and the maintenance of effective detoxification processes—is determined by its unique microarchitecture. Owing to its high regenerative capacity, the liver can restore structural integrity and functional reserves following partial loss of parenchyma caused by metabolic disorders, infectious diseases, surgical interventions, or toxic injury [1,2].

In recent decades, the need for hepatectomy has significantly increased due, on the one hand, to the rising incidence of extensive liver injuries and, on the other hand, to the growing number of transplantations performed at terminal stages of disease (e.g., primary and metastatic tumors, acute liver failure, cirrhosis, and decompensated disorders). According to global statistics, 34,694 liver transplantations were performed in 2021, increasing to 37,436 in 2022 [3,4]. In this context, and considering the implementation of the coordinated EU project “Mechanisms of liver regeneration and disease across scales: from molecules to cells and tissue” (REG_ORGatSCALE, 2023–2028), investigation of liver regeneration following partial hepatectomy (PH) is particularly relevant [5]. The classical experimental model for studying liver regeneration mechanisms is 70% PH in laboratory rats and/or mice [2,6].

Despite the remarkable regenerative capacity of the healthy liver, recent studies indicate that this capacity is impaired in two major clinical scenarios: (1) severe acute injury and (2) chronic injury accompanied by altered organ architecture and pronounced fibrosis, both of which may ultimately lead to liver failure [2,7,8]. Globally, drug-induced liver injury (DILI) accounts for approximately 50% of acute liver failure cases [8,9]. According to the FDA Adverse Event Reporting System (FAERS), which monitors drug safety, the largest number of reported DILI cases is associated with the uncontrolled use of paracetamol (acetaminophen, APAP) [10]. Clinical observations indicate that postoperative complications are often associated with insufficient or discoordinated liver tissue recovery, particularly in the presence of metabolic disorders, drug intoxication, oxidative stress, and drug–drug interactions [11,12]. These findings underscore the need to develop novel therapeutic strategies aimed at enhancing liver regeneration, including the targeted modulation of key signaling pathways, antioxidant defense systems, and the hepatic microenvironment.

During the initiation, progression, and modulation of the regenerative response, alongside intra- and extracellular signaling networks, changes in the redox state of hepatocytes play a significant role, mediated by the cellular pro-oxidant–antioxidant balance. During regeneration, proinflammatory cytokines (e.g., TNF-α), predominantly released by activated Kupffer cells, stimulate the production of reactive oxygen species (ROS). Within a certain concentration range, ROS participate in the regulation of intracellular signaling cascades involved in cell cycle progression and hepatocyte proliferation, including activation of NF-κB, ERK, and the NRF2/Keap1 pathways. However, disruption of the balance between the production and detoxification of reactive oxygen species, leading to oxidative stress, is regarded as a fundamental pathogenic mechanism underlying conditions associated with endogenous intoxication [11,12,13,14,15,16].

The search for alternative natural agents remains a promising strategy to reduce primary injury, modulate inflammation and oxidative stress, and stimulate regenerative processes following partial hepatectomy or toxic insults, such as acetaminophen-induced liver injury. Current pharmacological approaches are primarily aimed at mitigating the late consequences of acute damage; however, they do not always provide adequate support for regenerative processes and are limited by safety concerns and duration of use. In the field of antioxidant research, including its application in hepatology, microalgae are considered promising agents with multimodal mechanisms of action, as they are rich in diverse antioxidant bioactive compounds and have been extensively reviewed for their potential health benefits and redox-modulating properties [17,18]. In particular, freshwater unicellular algae of the genus *Desmodesmus* represent a valuable source of proteins, amino acids, pigments (including carotenoids), vitamins, and polyunsaturated fatty acids [19]. Furthermore, the growing emphasis on naturally derived bioactive compounds, owing to their favorable safety profile, biocompatibility, and sustainable production, positions microalgae as promising candidates for therapeutic application in drug-induced liver injury and partial hepatectomy settings [20,21,22].

Therefore, the aim of this study was to evaluate oxidative stress parameters, antioxidant defense mechanisms, and markers of liver injury and inflammation in rats subjected to partial hepatectomy under conditions of acetaminophen-induced toxic injury, as well as to assess the preventive effect of oral administration of a suspension of *Desmodesmus armatus* (*D. armatus*).

## 2. Materials and Methods

Unless otherwise specified, all reagents were of molecular biology grade and purchased from Merck Sigma-Aldrich (St. Louis, MO, USA).

### 2.1. Animals and Experimental Protocols

All animal procedures were approved by the Institutional Ethical Committee of Yuriy Fedkovych Chernivtsi National University, Ukraine (Protocol No. 1 from 4 April 2024) and conducted in accordance with national and international guidelines for the care and use of laboratory animals: European Convention for the Protection of Vertebrate Animals Used for Experimental and Other Scientific Purposes (Strasbourg, France, 1986), the provisions set out in the NIH Guide for the Care and Use of Laboratory Animals (Washington, USA, 2011) as well as the recommendations of the Bioethical Review of Preclinical and Other Scientific Research Conducted on Animals (Kyiv, Ukraine, 2006).

**Figure 1:**
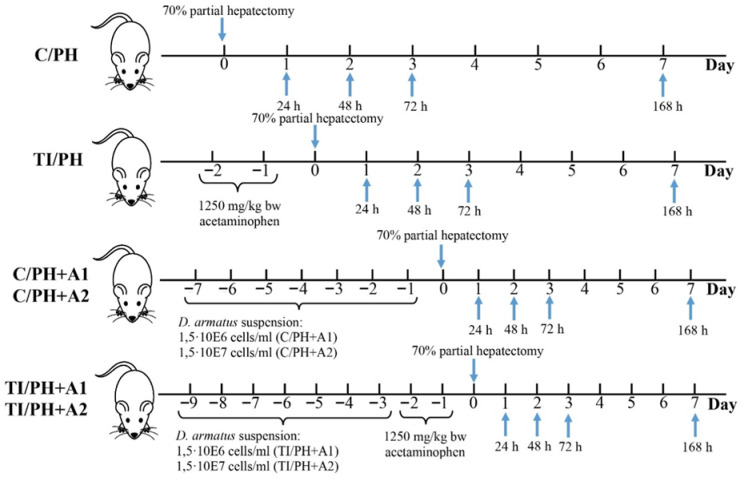
Schematic representation of the experimental design and sampling time points following partial hepatectomy. The analysis time points at 0, 24 h, 48 h, 72 h and 168 h are indicated by blue arrows. Group (C/PH)—control rats subjected to partial hepatectomy (PH) by two-thirds (2/3) liver resection; Group (TI/PH)—rats subjected to induction of acute toxic injury (TI) with acetaminophen (1250 mg/kg body weight), followed by two-thirds (2/3) liver resection (PH); Group (C/PH+A1)—rats pretreated for 7 days prior to PH with an oral suspension of *D. armatus* at a dose of 10 mL/kg body weight and a concentration of 1.5 × 10^6^ cells/mL; Group (C/PH+A2)—rats pretreated for 7 days prior to PH with an oral suspension of *D. armatus* at a dose of 10 mL/kg body weight and a concentration of 1.5 × 10^7^ cells/mL; Group (TI/PH+A1)—rats pretreated for 7 days with an oral suspension of *D. armatus* (10 mL/kg body weight; 1.5 × 10^6^ cells/mL), followed by induction of acetaminophen-induced liver injury and subsequent partial hepatectomy (PH); Group (TI/PH+A2)—rats pretreated for 7 days with an oral suspension of *D. armatus* (10 mL/kg body weight; 1.5 × 10^7^ cells/mL), followed by induction of acetaminophen-induced liver injury and subsequent partial hepatectomy (PH).

Experiments were conducted on male white outbred rats weighing 140–160 g and aged 140–150 days. Animals were housed in groups of three per cage prior to partial hepatectomy and individually after surgery, with ad libitum access to pre-sterilized water. Throughout the experimental period, rats received a standard balanced diet. Housing conditions were maintained at a controlled temperature of 22 ± 2 °C, with filtered air, 40–60% relative humidity, and a 12 h light/dark cycle [23]. Prior to the start of the study, rats were acclimated to the laboratory environment for 7 days, during which they had free access to a standard diet and water under the same light/dark cycle. The experimental design (Figure 1) included the following groups: Group (C/PH)—control rats subjected to partial hepatectomy (PH) by two-thirds (2/3) liver resection; Group (TI/PH)—rats subjected to induction of acute toxic injury (TI) with acetaminophen (1250 mg/kg body weight), followed by two-thirds (2/3) liver resection (PH); Group (C/PH+A1)—rats pretreated for 7 days prior to PH with an oral suspension of *D. armatus* at a dose of 10 mL/kg body weight and a concentration of 1.5 × 10^6^ cells/mL; Group (C/PH+A2)—rats pretreated for 7 days prior to PH with an oral suspension of *D. armatus* at a dose of 10 mL/kg body weight and a concentration of 1.5 × 10^7^ cells/mL; Group (TI/PH+A1)—rats pretreated for 7 days with an oral suspension of *D. armatus* (10 mL/kg body weight; 1.5 × 10^6^ cells/mL), followed by induction of acetaminophen-induced liver injury and subsequent partial hepatectomy (PH); Group (TI/PH+A2)—rats pretreated for 7 days with an oral suspension of *D. armatus* (10 mL/kg body weight; 1.5 × 10^7^ cells/mL), followed by induction of acetaminophen-induced liver injury and subsequent partial hepatectomy (PH). Five animals were included in each group at each time point. At the end of the experiment, animals were humanely euthanized by decapitation under light ether anesthesia, in accordance with the European Convention for the Protection of Vertebrate Animals Used for Experimental and Other Scientific Purposes (1986) and Directive 2010/63/EU of the European Parliament. This method was selected to allow rapid tissue collection and to minimize potential interference of anesthetic agents with mitochondrial respiratory chain enzymes. Blood samples were collected immediately after decapitation and centrifuged at 3000 rpm for serum separation and subsequent biochemical analyses. After confirmation of death, liver tissue was rapidly excised and processed at ice-cold temperature.

Experimental analyses were performed at 0 h (preoperative period), 24 h (initiation phase of liver regeneration), 48 h (active proliferation phase), 72 h (termination phase), and 168 h (late phase) after partial hepatectomy, as shown in Figure 1.

Partial hepatectomy was performed in the morning under fasting conditions and general anesthesia according to the method of Mitchell and Willenbring, which involves sequential ligation and aseptic resection of the left lateral (approximately 26% of liver mass) and median (approximately 40% of liver mass) lobes, corresponding to 2/3 of the total liver tissue [3]. The surgical procedure was carried out exclusively using sterilized instruments after quartz sterilization of the operating room. Sterile surgical silk (LLC “Igar”, Kyiv, Ukraine) was used for liver tissue ligation, and sterile surgical suture material “Kapron B” (SPE “Biopolymer”, Poltava, Ukraine) was used for continuous internal and external sutures. Anesthesia was induced with sodium thiopental (40 mg/kg, i.p).

Experimental acute liver toxicity was produced by oral administration of acetaminophen (APAP) at 1250 mg/kg body weight. The compound was prepared as a suspension in 2% starch gel and administered via intragastric gavage in a constant volume of 1 mL per animal. Prior to administration, the mixture was carefully homogenized to ensure consistent dispersion of APAP and precise dosing. Delivery was performed using a stainless steel feeding needle to ensure full administration of the calculated amount. The treatment was applied once daily for two days during the last two days before PH. This dosing regimen has been previously validated as effective for inducing APAP-related hepatotoxicity in experimental settings [24,25].

### 2.2. Microalgae Preparation and Administration

An algologically pure culture of the green microalga *Desmodesmus armatus* (Chod.) Hegew. was used in this study. The strain was obtained from the culture collection of the M.G. Kholodny Institute of Botany, National Academy of Sciences of Ukraine (IBASU-A), and is maintained in the collection of the Department of Biochemistry and Biotechnology, Yuriy Fedkovych Chernivtsi National University.

**Figure 2:**
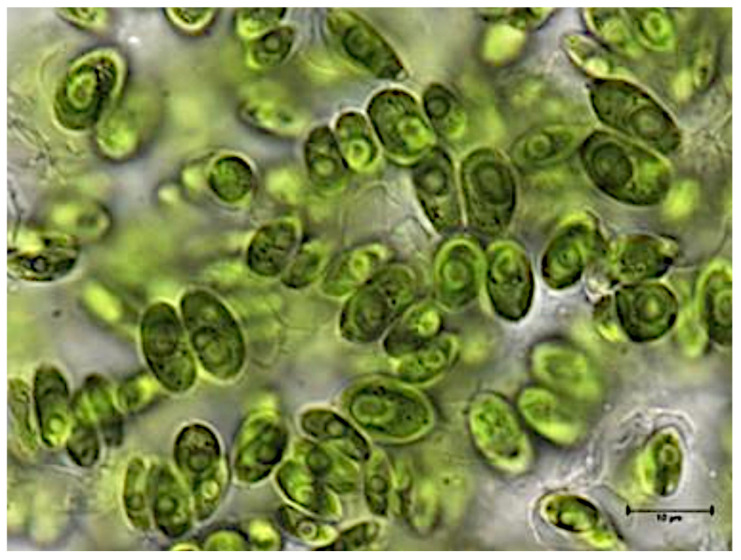
Photomicrograph of *Desmodesmus armatus* (Chod.) Hegew. Representative image obtained in the present study. Scale bar = 10 µm.

*D. armatus* is a freshwater species forming coenobia composed of 2, 4, or occasionally 8 cells arranged in linear or slightly alternating series. The cells are elongated- or oval-cylindrical, fused along up to two-thirds of their length, and typically rounded at the poles. They may bear small spines and longitudinal ribs, which can be continuous or interrupted and are present on all or only the median cells (Figure 2). Cell dimensions range from 7–15 µm in length and 3–6 µm in width [26].

The key characteristics for selecting an algal species include cell size, biomass growth rate under laboratory cultivation conditions, resistance to fluctuations in environmental parameters, nutritional value, and the absence of toxic compounds in the biomass composition. Previous studies by the authors have demonstrated the suitability of *D. armatus* as a feed and protective organism for various zooplankton species [27,28]. The biomass of *D. armatus* is characterized by a composition of approximately 50% proteins, up to 30% lipids, and about 20% carbohydrates. The amino acid profile comprises 17 proteinogenic amino acids [19,29]. In addition, the biomass contains chlorophylls and carotenoids. Analysis of the carotenoid fraction showed that primary carotenoids predominate, including zeaxanthin, lutein, and β-carotene, whereas minor amounts of astaxanthin, canthaxanthin, and esters of adonixanthin and astaxanthin have also been detected [19]. Moreover, it has been established that the biomass of *D. armatus* contains polysaccharides composed of monosaccharides such as mannose, rhamnose, glucuronic and galacturonic acids, arabinose, and fucose [21].

The culture of *D. armatus* was maintained in an aqueous minimal medium containing calcium (50–100 mg·L^−1^), magnesium (20–50 mg·L^−1^), potassium (2–20 mg·L^−1^), sodium (2–20 mg·L^−1^), fluorides (<1.2 mg·L^−1^), and iodine (<0.5 μg·L^−1^). Cultivation was performed in a climate-controlled chamber under a 16 h light/8 h dark photoperiod with fluorescent illumination (2500–4000 lx) at 24 ± 2 °C. Cells were grown to the stationary phase (21 days). The initial cell density was adjusted to 1.5 × 10^8^ cells/mL. Cell numbers were determined using a Goryaev counting chamber under a trinocular microscope ES-4120 (Micromed Evolution, Kyiv, Ukraine) at 100× magnification. Serial dilutions were prepared to obtain final concentrations of 1.5 × 10^7^ and 1.5 × 10^6^ cells/mL. Prior to administration, *D. armatus* suspensions were subjected to sterile ultrasonic disintegration using an Ultrasonic Cleaner CE-7200A (Guangdong Xinyi Ultrasonic Equipment Co., Ltd., Qingyuan, China) for 3 min at 50/60 Hz. The resulting biomass suspension was administered orally at 10 mL/kg body weight for 7 days before partial hepatectomy (groups C/PH+A1 and C/PH+A2) and before induction of acetaminophen-induced liver injury in partially hepatectomized rats (groups TI/PH+A1 and TI/PH+A2).

### 2.3. Biochemical and Inflammatory Rat Serum Markers

Serum alanine aminotransferase (ALT) and aspartate aminotransferase (AST) activities, as well as total and direct (conjugated) bilirubin concentrations, were measured using an automated biochemical analyzer (Biochem FC-120, High Technology Inc., North Attleboro, MA, USA) with commercial diagnostic reagent kits from the same manufacturer, according to the manufacturer’s instructions. Indirect (unconjugated) bilirubin levels were calculated as the difference between total and direct bilirubin.

Serum C-reactive protein (CRP) concentrations were determined using a sandwich-type solid-phase enzyme-linked immunosorbent assay (Rat CRP ELISA Kit, MyBioSource, San Diego, CA, USA), according to the manufacturer’s instructions. Absorbance was measured with a Stat Fax 2100 Microplate Reader (Awareness Technology Inc., Palm City, FL, USA), and concentrations were calculated according to the calibration curve provided by the manufacturer.

### 2.4. Histological Examination of Liver Tissue

For histopathological evaluation, liver specimens were immediately fixed in 10% neutral buffered formalin for 24 h at room temperature to preserve tissue architecture. Following fixation, samples were dehydrated through a graded ethanol series, cleared in xylene, and embedded in paraffin at 57–58 °C according to standard histological procedures [30]. Serial sections (5 μm thick) were obtained using a rotary microtome, mounted on glass slides, deparaffinized, rehydrated, and stained with hematoxylin and eosin for general morphological assessment.

Histological slides were examined using a Delta Optical Evolution 100 microscope equipped with planachromatic objectives (Delta Optical, Mińsk Mazowiecki, Poland). Representative images were acquired with an Olympus SP-550 UltraZoom digital camera (Olympus, Tokyo, Japan) mounted on the microscope.

Histological evaluation was performed using a semi-quantitative structured approach based on the assessment of key morphological parameters, including hepatocellular necrosis, inflammatory infiltration, steatosis, and disruption of hepatic architecture. Each parameter was scored using a four-point scale (0—absent, 1—mild, 2—moderate, 3—severe). For each liver section, 10 non-overlapping fields of view were analyzed, and scores were assigned for each parameter in every field. The mean score per section was calculated, followed by averaging across animals within each experimental group.

The assessment was performed in a partially blinded manner. Tissue processing and labeling were carried out by investigators aware of group allocation, whereas histological evaluation was performed by independent observers blinded to the experimental groups. Representative images were selected after comprehensive examination of all sections and fields of view (10 per section). For each experimental group, the predominant morphological pattern was identified based on consistent features observed across animals and fields. Images were then chosen to reflect this typical (modal) pattern rather than extreme or visually striking areas. Fields showing maximal or minimal alterations were intentionally avoided to ensure that the presented images accurately represent the overall histological state of each group.

### 2.5. Isolation of Liver Subcellular Fractions

Liver subcellular fractions were obtained by differential centrifugation. Fresh liver tissue was homogenized in ice-cold isolation buffer containing 0.25 M sucrose, 1 mM EDTA, and 10 mM Tris–HCl (pH 7.4) using a glass–Teflon homogenizer. The homogenate was centrifuged at low speed (800× *g* for 10 min at 4 °C) to remove nuclei and cellular debris. The resulting supernatant was subsequently centrifuged at high speed (9000× *g* for 10 min at 4 °C) to pellet the mitochondrial fraction, as previously described [31]. The mitochondrial pellet was washed twice and resuspended in EDTA-free Tris–HCl buffer to remove residual contaminants.

The microsomal fraction was isolated from the post-mitochondrial supernatant according to [32], based on Ca^2+^-induced aggregation of microsomal membranes followed by centrifugation at 10,000× *g* for 10 min at 4 °C. The resulting supernatant was collected as the cytosolic (post-microsomal) fraction. All isolation procedures were performed at 0–4 °C to preserve enzymatic activity and structural integrity of the subcellular components.

### 2.6. Assessment of ROS Production and Antioxidant Enzyme Activity (SOD and GPx)

The rate of mitochondrial superoxide radical (O_2_^•−^) production was determined using the nitroblue tetrazolium (NBT) reduction assay, based on the conversion of yellow NBT to blue diformazan by superoxide, with maximal absorbance at 540 nm [33]. Exogenous NADH was used to stimulate Complex I–dependent electron transport and to enhance detection of electron leakage–associated superoxide generation, which becomes more pronounced in structurally or functionally compromised mitochondria. Isolated mitochondria were suspended in isotonic phosphate buffer (pH 7.4), and NADH (18 mM) was added as a substrate to stimulate electron transport through Complex I. Mitochondrial samples were incubated at 37 °C for 10 min to allow substrate-driven O_2_^•−^ generation. Subsequently, 0.2% NBT solution prepared in Tris–HCl buffer (pH 7.4) was added, and the reaction mixture was incubated for an additional 5 min at 37 °C. The formed diformazan was extracted using a chloroform–dimethyl sulfoxide mixture (1:2, *v*/*v*), and absorbance was measured at 540 nm using a Cary 60 spectrophotometer (Agilent Technologies, Santa Clara, CA, USA). The reaction mixture (final volume: 1.0 mL) contained mitochondria at a protein concentration of 0.5 mg/mL. Protein content was determined by the Bradford method using bovine serum albumin as a standard. Blank samples containing all reagents except mitochondria were processed in parallel, and their absorbance values were subtracted from experimental readings to correct for non-specific NBT reduction. Results were expressed as the rate of NBT reduction, reflecting mitochondrial superoxide production.

Mitochondrial hydrogen peroxide (H_2_O_2_) levels were estimated using the sorbitol–xylenol orange (FOX) assay as previously described in [34]. The assay is based on the oxidation of Fe^2+^ to Fe^3+^ by hydrogen peroxide under acidic conditions, followed by formation of a Fe^3+^–xylenol orange complex. The resulting colored complex was quantified spectrophotometrically at 540 nm using a Cary 60 spectrophotometer (Agilent Technologies, Santa Clara, CA, USA).

Hydroxyl radical formation was estimated using the 2-deoxyribose degradation assay, based on the ability of ^•^OH to induce oxidative fragmentation of 2-deoxyribose, resulting in thiobarbituric acid–reactive substances (TBARS). The chromogen formed was measured spectrophotometrically at 532 nm by CARY 60 spectrophotometer (Agilent Technologies), and the results were expressed as the change in absorbance (ΔE × 10^2^) per 30 min per mg mitochondrial protein (arbitrary units) [35].

Mitochondrial and cytosolic superoxide dismutase (SOD; EC 1.15.1.1) activities were determined according to [36], based on the reduction in the rate of adrenaline auto-oxidation in alkaline medium. The assay relies on the ability of SOD to compete with adrenaline for superoxide radicals, thereby reducing the rate of adrenochrome formation. Changes in absorbance were monitored at 347 nm using a CARY 60 spectrophotometer (Agilent Technologies). One unit of SOD activity was defined as the amount of enzyme required to inhibit the rate of adrenaline autooxidation by 50% under the assay conditions. Results were expressed in arbitrary units per mg protein.

Glutathione peroxidase (GPx; EC 1.11.1.9) activity was evaluated by measuring the formation of oxidized glutathione (GSSG) during the reduction of hydrogen peroxide, as previously described [37]. The increase in absorbance associated with glutathione oxidation was recorded at 260 nm using the same spectrophotometer. Enzyme activity was expressed per mg protein.

### 2.7. Statistical Analysis

Statistical analysis was performed using GraphPad Prism 8.0.1 (GraphPad Software, San Diego, CA, USA). Normality of data distribution was assessed using the Shapiro–Wilk test. Differences between groups were evaluated using the non-parametric Mann–Whitney U test for pairwise comparisons, and the Kruskal–Wallis test followed by Dunn’s post hoc test for multiple group comparisons. A *p*-value < 0.05 was considered statistically significant. Data are presented as mean ± standard error of the mean (SEM). Some comparisons were prespecified as primary, while others were considered exploratory. Specifically, the primary comparisons included: (i) control vs. TI groups at baseline (0 h) and (ii) C/PH vs. TI/PH groups at corresponding post-hepatectomy time points. All additional within-group temporal comparisons (e.g., changes relative to preoperative values) and extended time-course analyses were considered exploratory.

## 3. Results

### 3.1. Biochemical Markers of Liver Injury and Systemic Inflammatory Response

**Figure 3:**
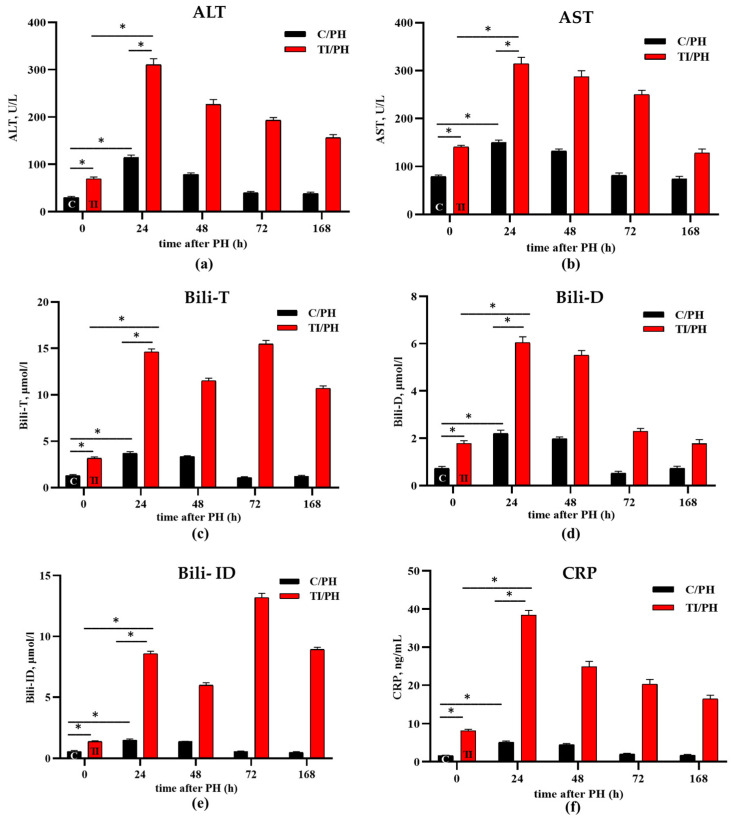
Serum biochemical markers of liver injury and inflammation in rats subjected to partial hepatectomy following acetaminophen intoxication. (**a**) Alanine aminotransferase (ALT) activity; (**b**) aspartate aminotransferase (AST) activity; (**c**) total bilirubin (Bili-T); (**d**) direct (conjugated) bilirubin (Bili-D); (**e**) indirect (unconjugated) bilirubin (Bili-ID); (**f**) C-reactive protein (CRP) concentration. C/PH: control rats (C) subjected to partial hepatectomy (PH); TI/PH: rats subjected to PH after induction of acute toxic injury (TI) with acetaminophen. Significant differences are indicated by * (*p* < 0.05) for the most notable values.

The analysis began with a comparison of injury markers at the initial stage of the experiment, prior to PH (time 0 in Figure 1), between control rats and rats with APAP-induced toxicity (TI). Here, as in the rest of the study, five animals were included in each group at each time point. Both aminotransferase levels, ALT and AST, were significantly higher in the TI group compared to the control group, showing increases of 2.3 ± 0.2-fold and 1.8 ± 0.1-fold, respectively (left pair of columns, time 0 in Figure 3a,b). Following PH, both ALT and AST levels increased in the C/PH group during the first 48 h and returned to preoperative values by 72–168 h (Figure 3a,b, black columns), reflecting a transient injury associated with normal regenerative processes.

In animals subjected to partial hepatectomy after acetaminophen intoxication (TI/PH), elevated ALT and AST levels persisted into the later stages of liver parenchyma recovery. ALT levels remained elevated up to 168 h (from 4.5 ± 0.3-fold at 24 h to 2.2 ± 0.2-fold at 168 h above preoperative values), whereas AST levels were increased up to 72 h (from 2.2 ± 0.1-fold at 24 h to 1.8 ± 0.1-fold at 72 h above preoperative values) compared with TI rats at 0 h (Figure 3a,b). When comparing the TI/PH and C/PH groups at the corresponding time points, persistently elevated ALT and AST levels were observed in the TI/PH group. For example, at 24 h after PH, ALT levels were 2.7 ± 0.2-fold higher, while AST levels showed a similar increase in the TI/PH group compared with the C/PH group (red vs. black columns in Figure 3a,b). Overall, the results indicate that APAP-induced toxicity leads to elevated baseline liver injury markers and, importantly, results in a more pronounced and prolonged increase in aminotransferase levels following partial hepatectomy compared to controls, highlighting delayed recovery dynamics in the TI/PH group.

Total serum bilirubin in C/PH animals increased 2.9 ± 0.1-fold at 24 h and remained elevated at 48 h (2.6 ± 0.1-fold) versus preoperative values, reflecting increases in both direct (~3.0-fold) and indirect (~2.6-fold) fractions (Figure 3c–e). In contrast, in the TI/PH group, total bilirubin levels remained elevated throughout the entire observation period (up to 168 h), with a marked peak at 72 h. This increase was primarily driven by the indirect bilirubin fraction (Figure 3e), indicating sustained impairment of bilirubin processing under combined toxic and surgical conditions. Direct two-group comparison of the C/PH and TI/PH groups at each time-point demonstrated consistently higher bilirubin levels in the TI/PH group, for example, 2.4 ± 0.1-fold higher at 0 h and 14.3 ± 0.8-fold higher at 72 h (Figure 3c).

To confirm the development of inflammatory processes under the studied conditions, serum CRP levels were assessed (Figure 3f). In the C/PH group, CRP levels increased slightly but significantly during the first 48 h of the regenerative process, compared with preoperative values. In contrast, in rats undergoing partial hepatectomy with acetaminophen-induced injury (TI/PH), CRP concentrations increased at 24 h and remained elevated throughout all stages of regeneration (up to 168 h), with the highest values recorded at 24 h (38.44 ± 1.2 ng/mL in TI/PH vs. 4.7 ± 0.4 in C/PH) (Figure 3f), reflecting an amplified systemic inflammatory reaction under combined toxic and surgical stress.

### 3.2. Morphological Changes in Liver Tissue

**Figure 4:**
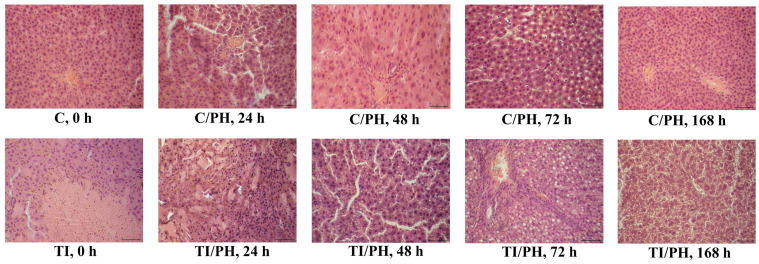
Histological structure of rat liver following partial hepatectomy (2/3 liver resection) after APAP-induced intoxication (hematoxylin and eosin staining, ×200). Experimental groups included: (C) control rats; (TI) rats with acute toxic injury; (C/PH) control rats subjected to partial hepatectomy (PH); and (TI/PH) rats with APAP-induced acute toxic injury that subsequently underwent partial hepatectomy (PH). Representative liver sections obtained before PH (0 h) and at 24, 48, 72, and 168 h after PH are shown. Scale bar: 100 µm.

In the preoperative period, the control group (C, 0 h) demonstrated preserved liver architecture: hepatic trabeculae were radially arranged, hepatocytes exhibited a typical polygonal shape with clearly defined nuclei, and no signs of inflammation or injury were observed (Figure 4). In contrast, histological examination of liver sections from animals with acetaminophen-induced toxic injury at 0 h (TI, 0 h) revealed disruption of lobular organization and radial arrangement of hepatic cords. Areas of hepatocyte necrosis with leukocytic infiltration were detected. Accumulation of an insoluble brown pigment, most presumably lipofuscin—one of the end products of lipid peroxidation—was observed predominantly in regions of necrotic changes (Figure 4), with a score of 2.8 in the TI group compared to 0.3 in the control group (Table 1, scores: 0 = absent, 1 = mild, 2 = moderate, 3 = severe, as described in Methods).

Subsequent histological analysis of the C/PH and TI/PH groups was performed at 24, 48, 72, and 168 h after partial hepatectomy. At 24 h after resection in the C/PH group, focal disorganization of hepatic trabeculae was observed, along with an increased number of binucleated hepatocytes, indicating proliferative activity, and dilation of the spaces of Disse. An increase in the relative volume of the stroma within interlobular triads was also noted, likely due to edema associated with venous congestion. In contrast, liver samples from TI/PH rats at this time point showed pronounced inflammatory and destructive changes in the parenchyma (Figure 4). The lobular architecture was disrupted, with marked polymorphonuclear leukocyte infiltration and evident foci of destruction (pale areas corresponding to necrotic regions at different stages, ranging from karyopyknosis to cellular detritus). These changes corresponded to increased scores for hepatocellular necrosis and inflammatory infiltration (2.9 for both parameters) in the TI/PH group compared to the corresponding C/PH group at 24 h (0.2 and 0.7, respectively). Edema of the connective tissue in the portal tracts was observed. Brown pigment inclusions, presumably lipofuscin, were already present in the TI group at 0 h and persisted throughout the observation period. An increase in the number of metaphase plates and binucleated hepatocytes was observed, indicating proliferative activity of hepatocytes.

Note, at 48 h after 2/3 liver resection in the C/PH group, in addition to binucleated hepatocytes, cells in mitosis with visible metaphase plates were observed, confirming active proliferation at this stage of parenchymal recovery. In contrast, rats of the TI/PH group at 48 h exhibited predominantly moderate microvesicular fatty degeneration of hepatocytes, mainly in peripheral zones of the hepatic lobules, likely reflecting APAP-induced toxicity. This was reflected by a steatosis score of 2.2, whereas no steatotic changes were detected in the corresponding C/PH group (score 0). Binucleated hepatocytes were also present, indicating proliferative activity of hepatocytes. Dilation of the spaces of Disse was visualized (Figure 4).

At 72 h, the regenerative process in the C/PH group was characterized by preservation of the trabecular structure of the hepatic parenchyma. Hepatocytes displayed moderately eosinophilic cytoplasm, and binucleated cells were still observed. Mild dilation of the spaces of Disse persisted against a background of sinusoidal congestion. In contrast, under conditions of partial hepatectomy following toxic doses of acetaminophen (TI/PH), marked mixed-type fatty degeneration (micro- and macrovesicular) was observed at this time point, affecting both the central and peripheral zones of the lobules. In some hepatocytes, nuclei were displaced toward the periphery, producing a “signet-ring” appearance. Disorganization (discomplexation) of the trabecular structure of the hepatic parenchyma was evident (Figure 4). These alterations were reflected by severe steatosis and moderate disruption of hepatic architecture scores in the TI/PH group at 72 h (2.9 and 2.0, respectively) compared with the corresponding C/PH group (0 and 0.2, respectively).

In the last observed time point, at 168 h post PH, the C/PH group demonstrated preserved trabecular-lobular architecture of the liver parenchyma. Stellate reticuloendothelial cells were clearly visualized between hepatocyte cords. In contrast, liver sections from the TI/PH group at 168 h showed disruption of trabecular organization, chaotic cellular arrangement, and indistinct cell contours in a significant number of hepatocytes. Granular and hydropic degeneration (reversible cellular swelling) was observed, along with dilation of the spaces of Disse. The histological pattern corresponded to massive liver injury with pronounced hepatocyte cytolysis, likely exacerbated by surgical resection on the background of APAP-induced toxic damage (Figure 4). Disruption of hepatic architecture was evident, with a score of 2.0 in the TI/PH group at 168 h, while no such changes were observed in the corresponding C/PH group (score 0).

### 3.3. Reactive Oxygen Species Production and Antioxidant Enzyme Activity (SOD and GPx)

**Figure 5:**
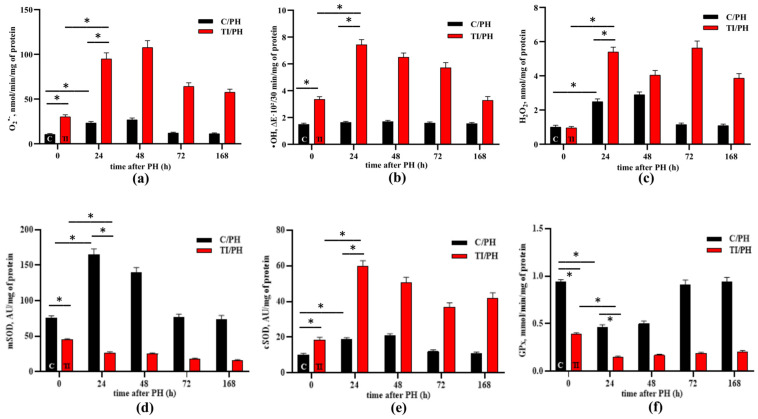
ROS production and antioxidant enzyme activity in the liver of rats after partial hepatectomy and acetaminophen-induced toxicity. (**a**) Superoxide anion (O_2_^•−^) production rate (nmol·min^−1^·mg^−1^ mitochondrial protein); (**b**) hydroxyl radical (^•^OH) formation rate (arbitrary units, calculated from the change in absorbance (ΔE × 10^2^) per 30 min per mg mitochondrial protein); (**c**) hydrogen peroxide (H_2_O_2_) levels (nmol·mg^−1^ mitochondrial protein); (**d**) mitochondrial superoxide dismutase activity (mSOD); (**e**) cytosolic superoxide dismutase activity (cSOD); (**f**) glutathione peroxidase (GPx) activity in the mitochondrial fraction. C/PH—the control (C) rats with partial hepatectomy (PH); TI/PH—the rats with PH after induction of acute toxic injury (TI) with acetaminophen. Significant differences are indicated by * (*p* < 0.05) for the most notable values.

Another damaging effect we assessed was the functionality of the mitochondrial electron transport chain, with particular focus on the capacity of Complex I to oxidize NADH. When electron flow through the respiratory chain is impaired, electron leakage can occur, leading to increased superoxide (O_2_^•−^) production. Therefore, we measured superoxide generation in isolated mitochondria following stimulation of electron transport with NADH (Figure 5a). Additionally, hydroxyl radical (^•^OH) rate, and hydrogen peroxide (H_2_O_2_) levels were assessed (Figure 5b,c).

While in control pre-intervention mitochondria low baseline level of (O_2_^•−^) was observed, APAP intoxication alone increased superoxide anion production and hydroxyl radical formation prior to hepatectomy (Figure 5a,b, time 0). Following partial hepatectomy, the C/PH group exhibited a transient increase in superoxide and H_2_O_2_ levels during the first 48 h, while ^•^OH production remained largely unchanged, indicating a controlled oxidative response during normal liver regeneration (Figure 5a–c, black columns).

In the liver mitochondria of rats subjected to partial hepatectomy following acetaminophen-induced injury (TI/PH), enhanced superoxide generation and elevated hydrogen peroxide levels persisted until the late stages of regeneration (up to 168 h). The most pronounced increase in superoxide production in the TI/PH group was observed at the early stages of liver regeneration, reaching 3.1 ± 0.3-fold at 24 h and further increasing at 48 h compared with the TI group at 0 h (Figure 5a, red columns). Additionally, hydroxyl radical production was significantly increased during the first 72 h in the TI/PH group (Figure 5b), further indicating persistent mitochondrial oxidative stress under combined toxic and surgical conditions.

At the next stage of the study, the activities of antioxidant defense enzymes, namely superoxide dismutase (SOD) and glutathione peroxidase (GPx), were evaluated. In the C/PH group, in which increased O_2_^•−^ and H_2_O_2_ levels were observed during the first 48 h of liver regeneration, both mitochondrial SOD (mSOD) and cytosolic SOD (cSOD) activities were significantly elevated compared with preoperative values (0 h). Specifically, mSOD activity increased 2.2 ± 0.1-fold at 24 h and remained elevated at 48 h, while cSOD activity increased ~1.9-fold at 24 h and reached 2.1 ± 0.2-fold at 48 h (Figure 5d,e; black columns).

In contrast, the TI/PH group exhibited a compartment-specific dysregulation of SOD activity. Mitochondrial SOD activity was consistently reduced throughout the recovery period, with the most pronounced decrease at later stages (72–168 h), suggesting impaired mitochondrial antioxidant defense (Figure 5d, red columns).

Conversely, in the cytosolic fraction of the TI/PH group, cSOD activity remained markedly elevated up to 168 h compared with preoperative values, with the most pronounced increase at 24 h (3.2 ± 0.3-fold) and a slightly lower elevation at 48 h (Figure 5e), likely reflecting a compensatory response to sustained oxidative stress.

For GPx, the pre-hepatectomy level was 58.6 ± 1.8% lower in the TI group than in the control group (Figure 5f, 0 h). Following partial hepatectomy, the C/PH group showed a transient decrease in GPx activity during the early phase (24–48 h), despite increased H_2_O_2_ levels, with recovery to baseline values at later time points (72–168 h) (Figure 5f, black columns). At 72 and 168 h, GPx activity returned to baseline (0 h) levels. In animals subjected to partial hepatectomy following acetaminophen-induced toxic injury (TI/PH), GPx activity showed a prolonged reduction, persisting up to 168 h compared with preoperative values. The decrease was most pronounced at 24 h (62.6 ± 3.7%) and remained substantial at later time points (~50%) (Figure 5f, red columns), indicating impaired antioxidant capacity under combined toxic and surgical stress.

### 3.4. Effect of Preventive D. armatus Administration on Hepatic Oxidative Stress After Partial Hepatectomy and Toxic Injury

To potentially improve the course of liver regeneration in rats following partial hepatectomy and toxic injury, a suspension of *D. armatus* microalgae was administered preventively at two concentrations: 1.5 × 10^6^ cells/mL and 1.5 × 10^7^ cells/mL. Since administration of the minimal culture medium used for *D. armatus* growth (experimental control) to animals of the C/PH and TI/PH groups did not produce any statistically significant changes in the studied parameters, preoperative values (C 0 h and TI 0 h) and the corresponding C/PH and TI/PH groups at each time point were used as reference controls.

**Figure 6:**
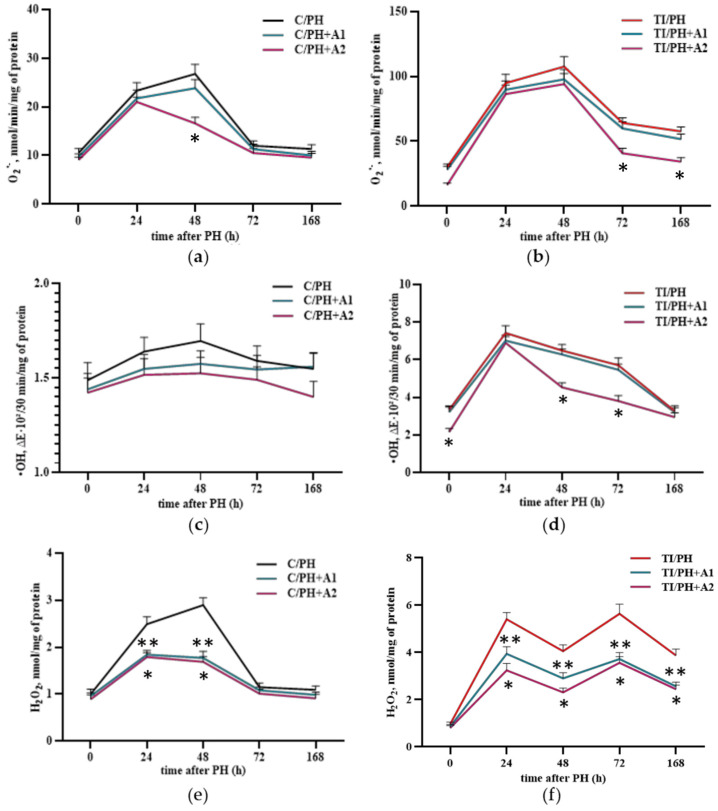
ROS formation in the mitochondrial fraction of liver cells of rats subjected to partial hepatectomy after acetaminophen intoxication under preventive administration of *D. armatus* suspension. (**a**) Superoxide radical formation rate in the C/PH group after pretreatment with low (C/PH+A1) and high (C/PH+A2) concentrations of *D. armatus*; (**b**) Superoxide radical formation rate in the TI/PH group after pretreatment with low (TI/PH+A1) and high (TI/PH+A2) concentrations of *D. armatus*; (**c**) Hydroxyl radical (^•^OH) formation rate in the C/PH group after pretreatment with low (C/PH+A1) and high (C/PH+A2) concentrations of *D. armatus*; (**d**) Hydroxyl radical (^•^OH) formation rate in the TI/PH group after pretreatment with low (TI/PH+A1) and high (TI/PH+A2) concentrations of *D. armatus*; (**e**) Hydrogen peroxide (H_2_O_2_) content in the C/PH group after pretreatment with low (C/PH+A1) and high (C/PH+A2) concentrations of *D. armatus*; (**f**) Hydrogen peroxide (H_2_O_2_) content in the TI/PH group after pretreatment with low (TI/PH+A1) and high (TI/PH+A2) concentrations of *D. armatus*. Statistical significance of the biological effects of *D. armatus* is indicated as follows: * for high concentrations and ** for low concentrations (*p* < 0.05).

In rats of the C/PH group, in which increased mitochondrial superoxide (O_2_^•−^) production was observed only at 24 and 48 h of regeneration (Figure 5a), preventive administration of *D. armatus* reduced O_2_^•−^ levels at 48 h. At the higher concentration (1.5 × 10^7^ cells/mL), O_2_^•−^ production decreased by 38 ± 6.4% compared with postoperative values at the same time point (Figure 6a). In the TI/PH group, preventive administration of *D. armatus* at both concentrations did not affect superoxide generation at 24 and 48 h. However, at later stages of regeneration (72 and 168 h), a decrease in O_2_^•−^ production was observed in rats receiving a high dose of *D. armatus*, with reductions of 36.4 ± 7.0% at 72 h, remaining similar at 168 h, compared with untreated TI/PH animals (Figure 6b).

Regarding hydroxyl radical (^•^OH) production, administration of the algae led to a reduction in ^•^OH levels in control rats; however, because baseline levels were low, this decrease was not statistically significant (Figure 6c). In the mitochondrial fraction of the TI/PH group, initially characterized by elevated ^•^OH levels, a significant decline in hydroxyl radical generation was observed at 48–72 h following administration of a high dose of *D. armatus*, with a reduction of (~30%). (Figure 6d). The low dose of *D. armatus* did not produce a significant effect.

Preventive administration of *D. armatus* reduced mitochondrial H_2_O_2_ levels in both experimental conditions, with a more pronounced effect in the TI/PH group. In the C/PH group, both concentrations led to a moderate reduction in H_2_O_2_ levels during the early phase of regeneration (24–48 h), indicating a protective effect under conditions of transient oxidative stress (Figure 6e). In contrast, in the TI/PH group, characterized by sustained elevation of H_2_O_2_, *D. armatus* administration resulted in a more substantial decrease across all time points, particularly at the higher concentration (Figure 6f). The strongest effect was observed at early stages (reductions of 40–43% at 24–48 h), while at later stages (72–168 h) the strong reduction persisted but showed less clear dose dependence.

**Figure 7:**
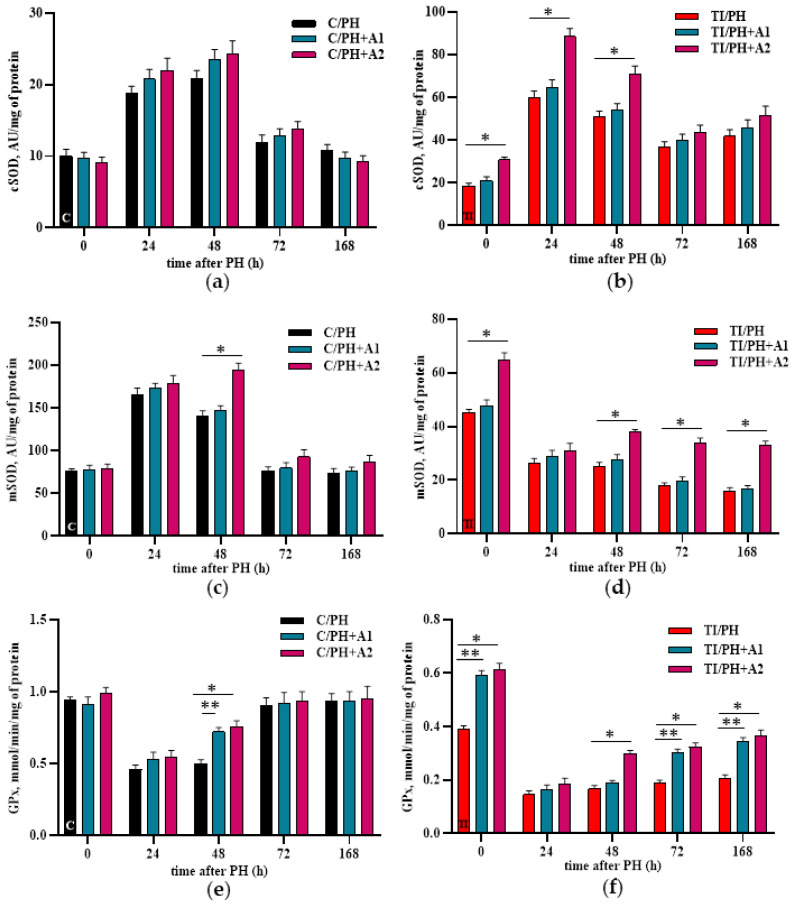
Superoxide dismutase (SOD) and glutathione peroxidase (GPx) activity in the liver of rats subjected to partial hepatectomy after acetaminophen intoxication under preventive administration of *D. armatus* suspension. (**a**) Cytosolic SOD activity (cSOD) in the C/PH group after pretreatment with low (C/PH+A1) and high (C/PH+A2) concentrations of *D. armatus*; (**b**) cytosolic SOD activity (cSOD) in the TI/PH group after pretreatment with low (TI/PH+A1) and high (TI/PH+A2) concentrations of *D. armatus*; (**c**) mitochondrial SOD activity (mSOD) in the C/PH group after pretreatment with low (C/PH+A1) and high (C/PH+A2) concentrations of *D. armatus*; (**d**) mitochondrial SOD activity (mSOD) in the TI/PH group after pretreatment with low (TI/PH+A1) and high (TI/PH+A2) concentrations of *D. armatus*; (**e**) GPx activity in the C/PH group after pretreatment with low (C/PH+A1) and high (C/PH+A2) concentrations of *D. armatus*; (**f**) GPx activity in the TI/PH group after pretreatment with low (TI/PH+A1) and high (TI/PH+A2) concentrations of *D. armatus*. Statistical significance of the biological effects of *D. armatus* is indicated as follows: * for high concentrations and ** for low concentrations (*p* < 0.05).

SOD activity was not markedly influenced by *D. armatus* administration. In the C/PH group, a significant increase in mitochondrial SOD (mSOD) activity was detected only at 48 h, whereas no significant changes in either mitochondrial or cytosolic SOD activity were observed at the other time points or at either concentration (Figure 7a,c). In the TI/PH group, characterized by already elevated SOD activity, further enhancement was observed following administration of *D. armatus* at the higher concentration. Cytosolic SOD activity increased only during the early stages of liver recovery (~40% at 24–48 h, Figure 7b). In contrast, mitochondrial SOD activity remained elevated from 48 to 168 h, with increases of 50.4 ± 7.1% at 48 h, rising further to ~90% at 72 h and ~108% at 168 h compared with the TI/PH group (Figure 7d).

GPx activity was not markedly affected by *D. armatus* under most experimental conditions. In the C/PH group, a limited response was observed, with a transient increase in mitochondrial GPx activity at 48 h following administration of both concentrations (Figure 7e). In contrast, *D. armatus* administration was more effective in the TI/PH group. The lower concentration increased GPx activity at later stages of regeneration for 61–66% at 72–168 h. The higher concentration induced a significant increase as early as 48 h (78.7 ± 14.4%), which remained elevated at later time points (~72–78%) (Figure 7f).

**Figure 8:**
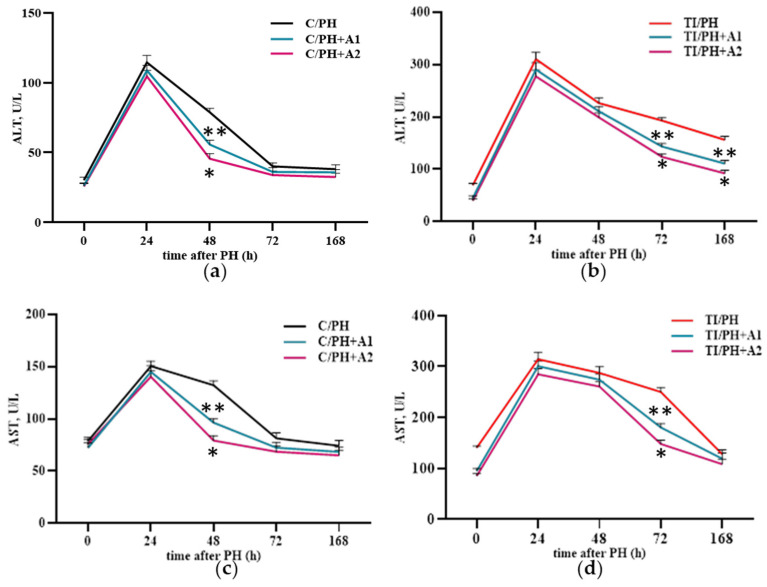
Aminotransferase activity in the serum of rats subjected to partial hepatectomy after acetaminophen intoxication under preventive administration of *D. armatus* suspension. (**a**) Cytolytic transaminases ALT activity in the C/PH group after pretreatment with low (C/PH+A1) and high (C/PH+A2) concentrations of *D. armatus*; (**b**) cytolytic transaminases ALT activity in the TI/PH group after pretreatment with low (TI/PH+A1) and high (TI/PH+A2) concentrations of *D. armatus*; (**c**) cytolytic transaminases AST activity in the C/PH group after pretreatment with low (C/PH+A1) and high (C/PH+A2) concentrations of *D. armatus*; (**d**) cytolytic transaminases AST activity in the TI/PH group after pretreatment with low (TI/PH+A1) and high (TI/PH+A2) concentrations of *D. armatus*. Statistical significance of the biological effects of *D. armatus* is indicated as follows: * for high concentrations and ** for low concentrations (*p* < 0.05).

Having established that preventive administration of *D. armatus* suspension is able to modulate prooxidant–antioxidant processes under conditions of partial hepatectomy and toxic injury, we further evaluated biochemical markers of liver damage and systemic inflammatory response under these experimental conditions. In the C/PH group, administration of *D. armatus* resulted in a reduction in serum ALT and AST levels at 48 h, indicating attenuation of transient hepatocellular injury during regeneration (Figure 8a,c). In contrast, in the TI/PH group, the protective effects were more pronounced at later stages of recovery. Pretreatment with *D. armatus* led to a significant decrease in ALT and AST levels at 72–168 h, with a stronger effect observed at the higher concentration (Figure 8b,d), suggesting improved recovery under combined toxic and surgical stress.

**Figure 9:**
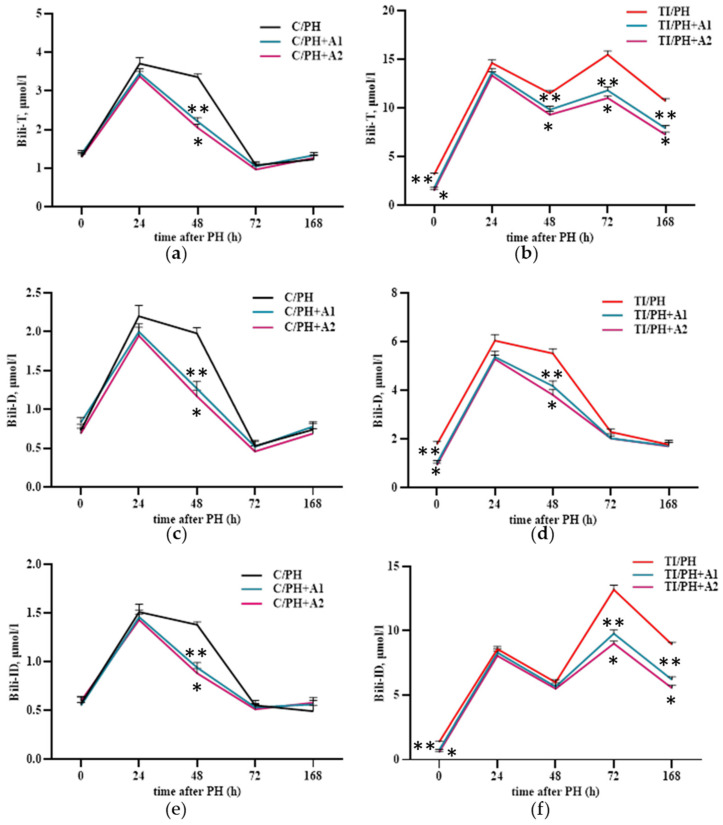
Serum bilirubin levels and its fractions in rats subjected to partial hepatectomy after acetaminophen intoxication under preventive administration of *D. armatus* suspension. (**a**) Total bilirubin (Bili-T) in the C/PH group after pretreatment with low (C/PH+A1) and high (C/PH+A2) concentrations of *D. armatus*; (**b**) Total bilirubin (Bili-T) in the TI/PH group after pretreatment with low (TI/PH+A1) and high (TI/PH+A2) concentrations of *D. armatus*; (**c**) Direct bilirubin (Bili-D) in the C/PH group after pretreatment with low (C/PH+A1) and high (C/PH+A2) concentrations of *D. armatus*; (**d**) Direct bilirubin (Bili-D) in the TI/PH group after pretreatment with low (TI/PH+A1) and high (TI/PH+A2) concentrations of *D. armatus*; (**e**) Indirect bilirubin (Bili-ID) in the C/PH group after pretreatment with low (C/PH+A1) and high (C/PH+A2) concentrations of *D. armatus*; (**f**) Indirect bilirubin (Bili-ID) in the TI/PH group after pretreatment with low (TI/PH+A1) and high (TI/PH+A2) concentrations of *D. armatus*. Statistical significance of the biological effects of *D. armatus* is indicated as follows: * for high concentrations and ** for low concentrations (*p* < 0.05).

Pretreatment with *D. armatus* did not markedly affect bilirubin levels in the C/PH group at 24 h. However, a significant effect was observed at 48 h, followed by a natural decline in bilirubin levels to pre-intervention values at 72 and 168 h. At 48 h, administration of both low and high concentrations of *D. armatus* resulted in a reduction in serum total bilirubin levels by 34–39% in the C/PH group, accompanied by decreases in both direct and indirect bilirubin fractions (Figure 9a,c,e). In the TI/PH group, *D. armatus* administration led to a consistent decrease in total bilirubin levels during the recovery period (48–168 h) at both tested concentrations (Figure 9b,d,f). For example, at 72 h, even the low *D. armatus* dose reduced total bilirubin levels from 15.48 ± 0.4 to 11.81 ± 0.3 µmol/L (Figure 9b).

Notably, at 48 h, the decrease in total bilirubin was primarily due to a reduction in the direct bilirubin fraction, whereas at later stages it was mainly attributable to decreases in the indirect bilirubin fraction (Figure 9b,d,f). The predominance of the reduction in direct (conjugated) bilirubin at 48 h suggests an early improvement in hepatocellular excretory function and bile flow during the active phase of liver regeneration. As direct bilirubin reflects canalicular secretion, its decrease may indicate restoration of hepatocyte function and reestablishment of biliary transport mechanisms at this stage. In contrast, the later decline in the indirect (unconjugated) bilirubin fraction likely reflects normalization of hepatic uptake, bilirubin conjugation capacity and overall metabolic stabilization of the regenerating liver parenchyma. Thus, the temporal shift in the predominant bilirubin fraction may indicate a sequential recovery of hepatocellular excretory and metabolic functions during liver regeneration.

**Figure 10:**
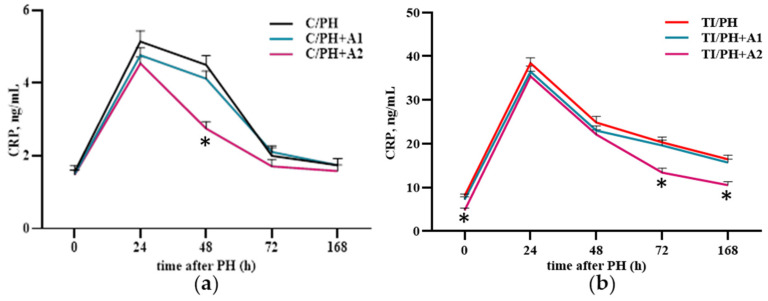
Serum C-reactive protein (CRP) concentration in rats subjected to partial hepatectomy after acetaminophen intoxication under preventive administration of *D. armatus* suspension. (**a**) CRP in the C/PH group after pretreatment with low (C/PH+A1) and high (C/PH+A2) concentrations of *D. armatus*; (**b**) CRP in the TI/PH group after pretreatment with low (TI/PH+A1) and high (TI/PH+A2) concentrations of *D. armatus*. Statistical significance of the biological effects of *D. armatus* is indicated as follows: * for high concentrations (*p* < 0.05).

The ability of *D. armatus* to improve serum CRP levels in animals is shown in Figure 10. In rats subjected to partial hepatectomy (C/PH), a significant reduction in CRP levels at 48 h postoperatively was observed only after administration of a high concentration of *D. armatus* suspension (C/PH+A2: 38.9 ± 5.0% reduction; Figure 10a). In TI/PH rats, CRP levels decreased at later stages of liver regeneration (72 and 168 h) following administration of a high dose of *D. armatus* (TI/PH+A2), with a reduction of 34.1 ± 7.8% at 72 h and a similar decrease at 168 h (~36%) (Figure 10b).

## 4. Discussion

In this study, we demonstrated that preventive administration of the microalga *Desmodesmus armatus* reduces oxidative stress, inflammation, and liver damage following partial hepatectomy in rats with acetaminophen-induced toxicity.

The degree of liver parenchymal recovery in rats subjected to partial hepatectomy following acetaminophen-induced toxic injury was assessed by monitoring serum ALT and AST activities, total bilirubin and its fractions, and C-reactive protein (CRP) levels. The increase in ALT and AST activities in the C/PH group during the first two days, with a peak at 24 h after surgery (followed by complete recovery within 72 h; Figure 3a,b), likely reflects the development of a moderate local inflammatory response triggered by tissue ligation and resection [16], which resolved within the expected timeframe. In contrast, the prolonged hyperenzymemia observed in the TI/PH group compared with C/PH indicates more pronounced postoperative parenchymal injury in the presence of prior APAP-induced toxicity.

The transient elevation of conjugated bilirubin within 48 h after resection in the C/PH group (Figure 3c–e) may be attributed to increased pressure in the intrahepatic bile ducts caused by portal tract edema associated with postoperative inflammation [38], leading to temporary intrahepatic cholestasis. The concurrent increase in unconjugated bilirubin may be attributed to impaired hepatocellular uptake of bilirubin from the sinusoidal blood, resulting from the substantially reduced functional liver mass and transient postoperative hepatic insufficiency. The normalization of bilirubin levels by day three (Figure 3c–e) may reflect rapid functional adaptation of the remnant liver, including restoration of hepatocellular transport and conjugation capacity, as well as activation of alternative degradation pathways, such as cytochrome P450–mediated N-oxidation reactions during early regeneration [23,39]. In the TI/PH group, sustained hyperbilirubinemia likely indicates pre-existing hepatocellular dysfunction compounded by reduced liver mass, impaired canalicular excretion, and limited glucuronidation capacity under APAP overload [40].

Alterations in biochemical markers were accompanied by inflammatory changes, as evidenced by elevated CRP levels (Figure 3f). In the C/PH group, the elevation of CRP within the first 48 h likely reflects an acute post-surgical inflammatory response. This transient increase may play a stimulatory and beneficial role by promoting tissue repair and initiating regenerative signaling, with CRP levels declining after 48 h as the inflammatory phase resolves. In contrast, persistently elevated CRP levels up to 168 h in the TI/PH group suggest impaired resolution of inflammation due to intoxication. This may be associated with dysregulated conversion of pentameric CRP (pCRP) to its monomeric pro-inflammatory form (mCRP) [41].

Histological analysis further revealed distinct morphological patterns depending on prior toxic injury. In C/PH animals, structural integrity was largely preserved, with evidence of proliferative activity, including binucleated hepatocytes and mitotic figures, and restoration of normal hepatic architecture by 168 h (Figure 4). Conversely, TI/PH rats exhibited persistent necrosis, leukocyte infiltration, and fatty degeneration, indicating sustained liver damage following the combined surgical and toxic insult. These findings are consistent with previous reports describing early hepatocyte necrosis following APAP toxicity mediated by the reactive metabolite NAPQI [42].

Beyond structural and biochemical alterations, maintenance of redox homeostasis is particularly important for proper liver function under toxic conditions. Increased production of O_2_^•−^ and H_2_O_2_ during the early regenerative phase (24–48 h) in both groups (Figure 5a–c) likely represents physiological redox signaling required for activation of proliferative pathways, including NF-κB, ERK, and NRF2 [13,14,15]. However, sustained overproduction of ROS, particularly ^•^OH, at later time points in the TI/PH group confirms a marked induction of oxidative stress and is consistent with other signs of pathological damage exacerbated by APAP-induced toxicity.

In the C/PH group, increased SOD activity during the first 48 h (Figure 5d,e) supports an adaptive response to elevated superoxide levels, promoting its conversion to H_2_O_2_ and subsequent detoxification. The normalization of ROS levels to baseline values by 72 h indicates restoration of redox homeostasis. In contrast, persistent suppression of mitochondrial SOD activity in the TI/PH group may result from oxidative or nitrosative inactivation of Mn-SOD (SOD2), including modifications of the Tyr34 residue within the active site [43,44,45,46]. The increased cytosolic SOD activity observed in TI/PH animals may reflect differential regulation of SOD isoforms, enhanced cytosolic superoxide production secondary to mitochondrial ROS leakage, or stress-induced activation of redox-sensitive transcriptional pathways (e.g., NRF2), rather than serving solely as a compensatory response to mitochondrial dysfunction.

Similarly, reduced GPx activity in both groups during early liver regeneration (Figure 5f) may allow maintenance of optimal H_2_O_2_ signaling concentrations [47]. However, prolonged suppression of GPx in TI/PH rats likely reflects glutathione depletion associated with NAPQI prior to surgery [48]. Despite the intrinsic regenerative capacity of the liver, combined surgical and toxic injury disrupts prooxidant–antioxidant balance and prolongs cytolytic and cholestatic syndromes. Notably, the time-dependent abnormalities observed following high doses of APAP combined with major liver resection may reflect persistent liver damage, combined physiological stress, or delayed recovery, rather than impaired regeneration alone.

Preventive administration of *D. armatus* suspension attenuated these disturbances by reducing ROS generation and enhancing SOD and GPx activities (Figure 6 and Figure 7), mostly with the higher concentration (1.5 × 10^7^ cells/mL) demonstrating greater efficacy. These effects may be attributed to the presence of carotenoids, polysaccharides, and potentially trace elements (Zn, Cu, Mn), as well as selenium within algal biomass, contributing to antioxidant defense mechanisms [21,29,49,50]. Importantly, improvements in redox status were accompanied by systemic benefits for liver, including reductions in ALT, AST, bilirubin fractions, and CRP levels (Figure 8, Figure 9 and Figure 10), indicating mitigation of cytolytic, cholestatic, and inflammatory syndromes.

In this study, we further explored the pathophysiology of APAP toxicity, particularly in the context of hepatectomy, a clinically relevant condition that may increase susceptibility to drug-induced liver injury. Multiple parameters were analyzed simultaneously, whereas in the published literature they have often been assessed only fragmentarily. Our findings support oxidative stress as a important molecular mechanism contributing to APAP-induced liver injury.

Although oxidative stress was monitored in this study, the lipid peroxidation, which is an important downstream consequence of reactive oxygen species (ROS) formation, was not directly assessed. Nevertheless, lipid peroxidation has previously been described in the context of APAP toxicity [24,42,51], and we propose that it may play a significant contributory role in the progression of cellular damage. Lipid peroxidation not only compromises membrane integrity but also generates highly reactive secondary products capable of covalently modifying proteins. Among the most biologically relevant lipid peroxidation-derived electrophiles are 4-hydroxynonenal (4-HNE), malondialdehyde (MDA), acrolein, and isoketals (γ-ketoaldehydes) [52,53,54]. These reactive aldehydes can form stable adducts with nucleophilic amino acid residues, thereby altering protein structure and impairing function. Such modifications have been documented in a variety of pathological conditions [51,55,56]. A variety of cellular functions have been shown to be affected by 4-HNE [55,57], and numerous functional proteins have been reported to be modified and damaged by 4-HNE [58,59,60], ranging from membrane receptors to cytochromes [61,62,63]. Accordingly, functional protein damage in APAP toxicity may result not only from the direct covalent binding of APAP or its reactive metabolite N-acetyl-p-benzoquinone imine (NAPQI), but also from additional secondary modifications mediated by lipid peroxidation products generated following oxidative membrane injury. However, the precise contribution of lipid peroxidation–derived protein adducts alongside APAP- and NAPQI-protein adducts remains unclear and warrants further investigation.

The hepatoprotective effects observed following administration of *Desmodesmus armatus* biomass can be tentatively attributed to its composite biochemical profile; however, these interpretations remain inherently limited by the use of a crude, non-fractionated preparation. The biomass is known to contain antioxidant constituents, including primary carotenoids (e.g., lutein, zeaxanthin, and β-carotene), which may contribute to the attenuation of reactive oxygen species and partial restoration of mitochondrial redox balance observed in this study. In addition, the presence of polysaccharides and other macromolecules may exert modulatory effects on inflammatory responses, potentially contributing to the reduction in systemic inflammatory markers. The relatively high protein and amino acid content may also support regenerative processes following partial hepatectomy. Nevertheless, given the lack of targeted compositional analysis and the absence of functional validation of individual fractions, any mechanistic interpretation should be considered preliminary. It remains unclear which specific components, if any, are primarily responsible for the observed biological effects, and whether these arise from direct antioxidant activity, indirect modulation of cellular pathways, or nonspecific metabolic support. Further studies employing fractionation approaches, molecular characterization, and pathway-level analyses are required to substantiate these hypotheses and to define the mechanistic basis of the observed hepatoprotective effects. Additional studies are needed to determine whether *D. armatus* is able to enhance liver regeneration. The currently available data suggest a more favorable environment for liver recovery, rather than directly demonstrating enhanced liver regeneration. Future studies should also explore alternative modes of *D. armatus* administration, as well as the isolation and characterization of the specific bioactive compounds responsible for the observed effects.

## 5. Conclusions

Our findings suggest that preventive administration of *Desmodesmus armatus* attenuates liver injury, oxidative stress, and inflammatory responses following partial hepatectomy complicated by acetaminophen-induced toxicity. These results identify *D. armatus* as a promising natural biomodulator with hepatoprotective potential. Further studies are warranted to clarify its underlying mechanisms of action and to evaluate its potential applicability in strategies aimed at improving liver functional reserves under conditions of toxic liver injury.

## Figures and Tables

**Table 1:** Semi-quantitative histological assessment of rat liver following partial hepatectomy (2/3 liver resection) after APAP-induced intoxication.

	Disruption of Hepatic Architecture	Hepatocellular Necrosis	Inflammatory Infiltration	Insoluble Brown Pigment Accumulation	Steatosis
C, 0 h	0 ± 0	0 ± 0	0 ± 0	0.2 ± 0.09	0 ± 0
TI, 0 h	2.8 ± 0.23 ^a^	2.7 ± 0.20 ^a^	2.6 ± 0.22 ^a^	2.8 ± 0.26 ^a^	0.1 ± 0.07
C/PH, 24 h	0.5 ± 0.12	0.2 ± 0.10	0.7 ± 0.13	0.3 ± 0.10	0.1 ± 0.06
TI/PH, 24 h	3.0 ± 0.22 ^b^	2.9 ± 0.22 ^b^	2.9 ± 0.21 ^b^	2.1 ± 0.22 ^b^	0.2 ± 0.09
C/PH, 48 h	0.3 ± 0.11	0 ± 0	0.4 ± 0.13	0.2 ± 0.10	0 ± 0
TI/PH, 48 h	2.5 ± 0.23 ^b^	1.8 ± 0.22 ^b^	2.2 ± 0.23 ^b^	0.7 ± 0.18	2.2 ± 0.23 ^b^
C/PH, 72 h	0.2 ± 0.12	0 ± 0	0.2 ± 0.11	0.2 ± 0.09	0 ± 0
TI/PH, 72 h	2.0 ± 0.19 ^b^	0.4 ± 0.13	1.7 ± 0.20 ^b^	1.5 ± 0.19 ^b^	2.9 ± 0.20 ^b^
C/PH, 168 h	0 ± 0	0 ± 0	0.2 ± 0.12	0.2 ± 0.10	0 ± 0
TI/PH, 168 h	2.0 ± 0.21 ^b^	0.3 ± 0.15	1.3 ± 0.18 ^b^	1.0 ± 0.17 ^b^	1.7 ± 0.21 ^b^

Experimental groups included: (C) control rats; (TI) rats with acute toxic injury; (C/PH) control rats subjected to partial hepatectomy (PH); and (TI/PH) rats with APAP-induced acute toxic injury that subsequently underwent partial hepatectomy (PH). (^a^) significant difference between the TI group and the control (C) group at 0 h; (^b^) significant difference between the TI/PH and C/PH groups at the corresponding time point (e.g., 24 h vs. 24 h).

## Data Availability

The original contributions presented in the study are included in the article. Further inquiries can be directed to the corresponding authors.
